# Changing antiretrovirals whilst viral load <50 copies/ml and relationship with CD4 count changes

**DOI:** 10.1186/1758-2652-13-S4-P24

**Published:** 2010-11-08

**Authors:** A Mocroft

**Affiliations:** 1 University College London Medical School, Royal Free Campus, Rowland Hill St, UK

## Purpose of the study

The frequency and reasons for switching antiretrovirals (ARVs) in patients on a fully suppressed cART regimen (viral load [VL] <50copies/ml) is not well described, nor is the effect of such a change on CD4 counts.

## Methods

6713 patients from EuroSIDA on cART with a confirmed VL<50 copies/ml were included; a regimen change was defined as >1 ARV change (occurring on the same day) for any reason whilst VL< 50 copies/ml. Baseline was defined as the first VL<50 copies/ml on cART; Kaplan Meier methods estimated the probability of ARV change and Cox proportional hazards models, stratified by centre, identified factors associated with ARV change. Mixed models were used to model the change in CD4 count after the first ARV change.

## Results

At baseline, the median CD4 was 414/mm^3^ (IQR 272—587). 1079 (16.1%) patients changed 1358 ARVs; 93 (8.6%) patients started an ARV they had previously taken. 224 (77.0%) of those starting an NNRTI (n=291) were previously naïve to NNRTIs, compared to 29 of 306 who started a PI or boosted-PI (9.5%). The incidence of changing ARVs was 11.8 per 100 PYFU (95% CI 11.1-12.5). At 1 year after baseline, 10.7% were estimated to have changed >1 ARV (95% CI 9.8-11.5). The most common reason for change was toxicity (n=521, 38.4%), followed by patient or physician choice (n=398, 29.3%). Table 1 shows the factors associated with changing ARVs.

**Table 1:** 

		Multivariate		
		RH	95% CI	P

Gender	Female versus Male	1.29	1.09-1.53	0.0038

Risk group	Heterosexual versus other	0.83	0.70-0.98	0.033

Basline	Per year later	1.32	1.27-1.38	<0.0001

Nucleoside pair	Zidovudine/lamivudine	1.00	-	-

	Didanosine/stavudine	1.94	1.49-2.53	<0.0001

	Stavudine/lamivudine	1.81	1.50-2.17	<0.0001

	Tenofovir plus 1	0.87	0.68-1.10	0.24

	Abacavir plus 1	0.62	0.46-0.83	0.0012

	Other not listed	1.06	0.80-1.40	0.68

Third drug	Single PI	1.00	-	-

	Boosted PI	0.46	0.36-0.58	<0.0001

	NNRTI	0.42	0.35-0.50	<0.0001

	Triple nucleoside	0.23	0.16-).35	<0.0001

HCV serostatus	Positive versus negative/unknown	0.80	0.67-0.96	0.017

After adjustment, changing ARVs was associated with an additional annual increase in CD4 counts of 9.3/mm^3^ per year (95% CI 5.7-12.9/mm^3^) compared to not changing ARVs. The increase was similar in patients who recycled ARVs compared to those starting an ARV to which they were naive, according to type of new ARV started (nucleoside, PI, boosted-PI or NNRTI), and number of new ARVs started (0, 1 or >2, p>0.05 all). Patients starting a new ARV class had higher increases in CD4 counts compared to those who changed ARVs but did not start a new class (8.0/mm^3^; 95% CI 0.2-15.8/mm^3^, p=0.044), although there was no differences between PI-containing or NNRTI-containing classes (p=0.54).

## Conclusions

Changing ARVs whilst virologically suppressed was due to patient/physician choice or toxicity, increased in frequency over time and was more common in patients taking a single PI-regimen or in stavudine-containing regimens. Patients who changed ARVs had a small but statistically significant boost to CD4 count levels, and the increase in CD4 was higher in those who changed to a new class of ARV.

